# P-Hint-Hunt: a deep parallelized whole genome DNA methylation detection tool

**DOI:** 10.1186/s12864-017-3497-9

**Published:** 2017-03-14

**Authors:** Shaoliang Peng, Shunyun Yang, Ming Gao, Xiangke Liao, Jie Liu, Canqun Yang, Chengkun Wu, Wenqiang Yu

**Affiliations:** 1 School of Computer Science, National University of Defense Technology, Changsha, China; 2 Faculty of Life Sciences, University of Manchester, Manchester, UK; 3 EpiRNA Lab, Institues of Biomedical Sciences, Fudan University, Shanghai, China

**Keywords:** DNA methylation detection, Whole genome, Parallelized algorithm, Xeon Phi, Tianhe-2

## Abstract

**Background:**

The increasing studies have been conducted using whole genome DNA methylation detection as one of the most important part of epigenetics research to find the significant relationships among DNA methylation and several typical diseases, such as cancers and diabetes. In many of those studies, mapping the bisulfite treated sequence to the whole genome has been the main method to study DNA cytosine methylation. However, today’s relative tools almost suffer from inaccuracies and time-consuming problems.

**Results:**

In our study, we designed a new DNA methylation prediction tool (“Hint-Hunt”) to solve the problem. By having an optimal complex alignment computation and Smith-Waterman matrix dynamic programming, Hint-Hunt could analyze and predict the DNA methylation status. But when Hint-Hunt tried to predict DNA methylation status with large-scale dataset, there are still slow speed and low temporal-spatial efficiency problems. In order to solve the problems of Smith-Waterman dynamic programming and low temporal-spatial efficiency, we further design a deep parallelized whole genome DNA methylation detection tool (“P-Hint-Hunt”) on Tianhe-2 (TH-2) supercomputer.

**Conclusions:**

To the best of our knowledge, P-Hint-Hunt is the first parallel DNA methylation detection tool with a high speed-up to process large-scale dataset, and could run both on CPU and Intel Xeon Phi coprocessors. Moreover, we deploy and evaluate Hint-Hunt and P-Hint-Hunt on TH-2 supercomputer in different scales. The experimental results illuminate our tools eliminate the deviation caused by bisulfite treatment in mapping procedure and the multi-level parallel program yields a 48 times speed-up with 64 threads. P-Hint-Hunt gain a deep acceleration on CPU and Intel Xeon Phi heterogeneous platform, which gives full play of the advantages of multi-cores (CPU) and many-cores (Phi).

## Background

Epigenetics focus on the problem that why lives with same DNA sequences have different characters. Epigenetics is an important part of the genetics. There are two kinds of information in the genome: the explicit information in DNA sequence and implicit information hiding in the combination of bases. These implicit information control some characters of lives [1]. Although researchers have already started the study of epigenetics for decades, they have not reached the purpose of revealing the laws of epigenetics [2, 3]. Especially for research on DNA methylation, the research about it has been lasted about 20 years. But what we know about DNA methylation is pretty preliminary.

Whole genome DNA methylation detection is one of the most important part of epigenetics research. It is supposed to have a great effect on cancers and tumors, and even be involved in the senility of human. In addition, it is believed that in medical aspects, DNA methylation may have a strong relationship with diabetes and immunological diseases [4–6]. Especially for cancers, the abnormity of DNA methylation in some specific area of DNA has a great significance of carcinogenesis. For now, the main method to detect whole genome DNA methylation is mapping the target sequences, after bisulfite treatment, back to the reference genome, and tell the differences [7–10]. But in practical research, mapping tools that are widely used may fail to find the accurate map or they cannot find the map quickly. The root cause of finding inexact map is that these tools only use bisulfite treated sequences, which have already been changed. Also, with the rapid development of biotechnology, the dataset of DNA sequences to carry out the methylation analysis become larger and larger, which is supposed to lead to a much time-consuming work of finding maps.

For example, BSMAP [11] is a representative tool to detect the whole genome DNA methylation. But with the research go deeper and the sequence data go bigger, there are two main challenges ahead of us: the accuracy and the speed. BSMAP maps the bisulfite treated sequences directly to the whole genome reference. However, because the sequences are treated by bisulfite, some of them have been changed, which will bring mistakes during the mapping operator. An efficient way to solve this problem is to use the sequences of one end with bisulfite treatment and another end without bisulfite treatment. This is a relatively newer and harder area which may lead to better results. Another challenge is the low-speed problem. Because BSMAP only supports multi-threads on single computing node, as the data of sequences grows fast, one sample from a single biological experiment could generate 1 TB sequence data, which may take several months for general computing platform using BSMAP to process them. Hence, there is a strong demand of whole genome DNA methylation detection tool, which can run with multiple threads on platform with multiple computing nodes.

**Fig. 1:**
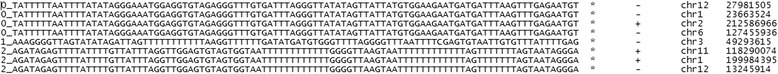
Target sequences format. The number before each sequence is an ID which must be consistent on the same chromosome. The third column represents the positive or negative chains of DNA. The fourth column represents the chromosome this sequence is located in. The last column represents the coordinates of the sequence in the original reference

Nowadays, there are mainly two parts of DNA methylation detection: biological sequencing by experiment and computer simulation. After the biological experiment, we can acquire the sequences with assistant location information. The samples of paired-ends sequences information are shown in Fig. 1.

According to the location information in the last three columns, we could acquire the mapping range to narrow the suspected area, as well as avoiding the cost of aimless mapping.

**Fig. 2:**
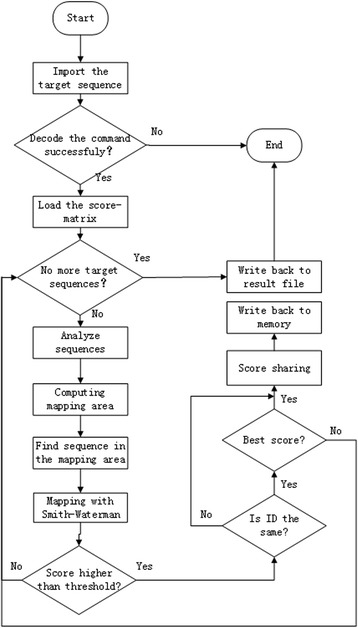
The whole pipeline of P-Hint-Hunt. The pipeline is clear enough. We need to be aware of that the only dependency of the data is that target sequences with the same ID must be processed within the same thread to achieve the best result. Therefore, when a sufficiently high score is obtained, we still make a corresponding judgement before we process the next sequence


After locating specific areas of mapping in the reference sequence, we use the Smith-Waterman algorithm [12] to map target sequence to the reference sequence in a best matching mode by a local backtracking strategy. Then, we could acquire the most similar sequence information and location information and give out the DNA methylation detection result of the human genome by scanning these information. The whole pipeline of the program is shown in Fig. 2. The pseudocode of local backtracking shown in Algorithm 1.

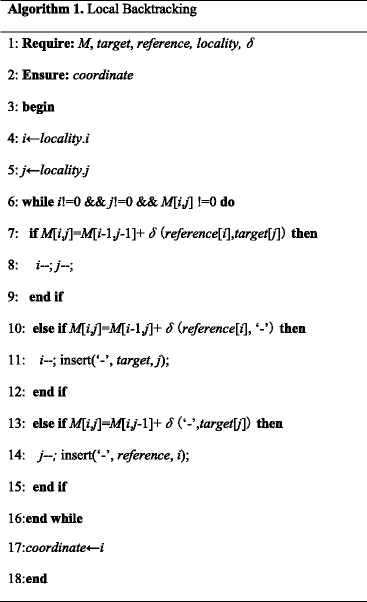


In our work, we use the sequences of one end with bisulfite treatment and another end without bisulfite treatment, which is a relatively newer and harder area may lead to better results, to solve the problem of inaccuracy. First, we use OpenMP to design a fine-grained multi-threads approach to massively parallelize the complex tasks of big data to achieve a good performance. Then, we propose a new parallel Smith-Waterman algorithm to map the sequence precisely in the range of 1000 bp around the preliminary location. The new algorithm we proposed is removing whole backtracking step of original Smith-Waterman method by sacrificing a small amount of space effectiveness to in exchange for higher performance gains. Specifically, we record the best source direction for each matrix element in the matrix score calculation step. Based on this information, we can backtrack directly without the need for redundant branch judgments. This is a more efficient method of determining the best matching position.

After the data screening and deletion of PCR duplicates, we choose the best mapping result for DNA methylation analysis. In order to promote the performance, we design a centralized looping IO mode and build an index of input data to effectively avoid the extra overhead caused by frequent IO operation. Further, we develop the program to exploit multi-level parallelism using multi-threads and multi-processes, which could be adjusted flexibly according to various computing platform. And the Intel Xeon Phi coprocessors are also used to cooperate with CPUs to achieve a faster computing speed. At last, we evaluate our method on Tianhe-2 supercomputer. The results show that the correctness of program is guaranteed both for serial version and parallel version. The speed-up is 47 times with 64 threads, and the speed-up of multiple nodes is nearly linear.

## Methods

After the authentication, we find that Hint-Hunt could figure out the methylated information accurately based on the mapping in a specific area, but when facing the computation of big data like the whole human genome, there are still problems like too much time consumption, especially when we use the sequence data with more cover layers to reach a large scale. In order to solve these problems, we design the parallel version of Hint-Hunt, the P-Hint-Hunt, to optimize the partition of task, the establishment of index and the function of IO with multi-levels parallelism.

### The optimization of multi-threads

Because of the massive data, nearly 3 TB, the serial program could not meet the requirements of running time. Therefore, we optimize the serial program to the parallel version and implement the use of multi-threads based on OpenMP, reaching a speed up of 47 times using 64 threads.

There are two alternatives to implement multi-threads: the coarse parallelism of chromosome level and the fine-grained parallelism of target sequence level. The coarse parallelism means we could use one thread to process the data from each chromosome, 25 threads in total. This approach is easier to implement as there is no need to consider the task division. But because of the difference of the amount of target sequences located on the same chromosome, there will be huge difference of loads on different threads, which will lead to severe load imbalance among these threads. On the contrary, the fine-grained parallelism version could modify the number of threads dynamically according to the amount of data, and reach the load balance to the maximum extent.

Before we start, we analyze the data dependency and program structure. The only dependency of the data to be processed is that target sequences with the same ID must be processed within the same thread to pick out the best result. Except this, there is no any other data dependence among the target sequences. The analysis of the program structure divide the program to two main parts: the I/O part and the process of characters. The second part takes the most of running time, which is needed to be parallelized eagerly.

The implementation consists of three main parts. (1) Loading data and pre-classification. According to the input data, with an order of ID number, we classify all of the input data to avoid the bad influence caused by data dependence. We set the same ID number for target sequences, and divide the indexes to different threads equally basing on the amount of target sequences in each index. This procedure insures the correctness of the program and keeps the load balance, which also reduces the running time. (2) Concentrated loop IO operation help to control the usage of memory. In the serial program, the program firstly reads the target sequence file line by line and then computes and processes. After these process operations, the program writes the result back to result arrays and finally writes the results back to result files when all the processes are over. The multi-threads structure needs to import the data into memory first to help task partition and establishment of indexes, but the frequent IO operations are barriers for pipeline execution on hardware. So we use concentrated loop IO operation (import two million lines of sequence data once upon a time) to avoid the performance decrease caused by frequent IO operation and help control the usage of memory at the same time. (3) The safety of multi-thread running. In the programming of multi-thread program, there are both shared space and private space in every thread, so first we have to classify the shared parameters and the private parameters. In the program, the array of reference sequence and the array of overall methylated position are shared by all the threads. In addition, to make sure the safety of multi-thread running, we use the thread-safe functions instead of the general function. Take the function in C++ base for example, “strtok” is not thread-safe, which means it may occur errors when running several threads sharing memory. So we use the function “strtok_r” instead.

### The optimization of multi-processes

Using the multi-cores on single node could not meet the requirement of time consumption when the data scale big enough. To make full use of the structure of supercomputer, the multi-processes version of program could take the advantages of multiple computing nodes to accelerate the running time extremely.

First, the program calculates the amount of data needed to be divided to each process according to the number of processes. During the division of tasks, we need to insure that target sequences with exactly the same ID number must be divided to the same process. The processes communicate with each other to acquire the start and end position of their tasks, and then start to process data, where the load balance have been insured among all processes.

### The cooperation of Intel Xeon Phi coprocessor and Intel CPUs

Nowadays, more and more supercomputers provide powerful computing capacities base on a heterogeneous architecture. Intel Xeon Phi coprocessor and GPUs are added to the traditional computer architecture to seek for an increase of computing scale, and the former is more powerful in parallel computing and scientific computing. Take the Tianhe-2 super computer for an example, each of its computing nodes have two Xeon E5 2692 processors and three Intel Xeon Phi coprocessors. So we optimize the parallel program especially for the architecture of super computers.

The main challenge of programming on coprocessors is the usage of memory on coprocessors. Because the coprocessors cannot access to the main memory as CPU does, so the memory remains to be idle on each coprocessor in practical is nearly 6GB. However, the memory allocating on CPUs is about 28GB. Therefore, we use CPU to control the data transformation between main memory and memory on coprocessors. We use 112 threads of 56 cores on the Intel Xeon Phi coprocessors to process 100 million lines (this amount is calculated by the memory available) of target sequences each time, and then carry out data transformation between these two kinds of memory.

About the cooperation of Intel Xeon Phi coprocessor and Intel CPU, we find that the computing capability of three coprocessors using 56 cores each is nearly the same as two CPUs with 12 cores each after the tests. So, we divide the target sequence file into 12 parts and distribute two parts of them to each coprocessor and three parts of them to each CPU processor. The dynamic distribution of tasks makes sure the load balance between CPUs and coprocessors and makes the full use of all the computing resources.

### The pre-process of reference sequence file

The reference sequences are stored in the file in the format of 50 bp per line, which is imported line by line. Therefore, when the input data become large, the time of IO could be very substantial. In order to solve this problem, we use a pre-process of reference sequence file to remove the line break symbols in it and make sure the location of each base remain the same. After this operation, the program could import the reference sequence file in a very short time. It only takes 20–30 s rather than original several hours.

### The optimization of data structure and better usage of memory

At the very beginning of our program, in order to record the methylated or non-methylated information, we use structural arrays to record the start and end mapping position, score, etc. Take one of several input files for example, there are 9,242,825 lines of sequence in the target sequence file, and every one of the sequences needs the space of 212 Bytes. That makes the total space needed for the target sequences to be used as a buffer are 212B × 9242825 = 1.825 GB. In addition, another 3GB are still necessary to store the human genome reference sequences because the length of reference is 3G. In order to store the methylated and non-methylated information of each base in the references, we need extra 3G × 8B = 24GB memory space. While, the target sequence file in this example is the minimum one, which means that there could be a need for 70 × 1.825GB + 3GB + 24GB = 154.75 GB memory space for the largest target sequence file consisting 650 million lines of sequences, nearly 70 times bigger than the first example. The demand for memory is obviously beyond the capability of common computing station, and will keep increasing as the target sequence file increases.

Aiming at solving these problems, we optimize the data structure to minimize the memory allocation. When storing the information of methylated and non-methylated, the program uses a buffer in each thread, which is allocated temporarily, rather than a permanent piece of block in the memory. And each thread then releases the buffer after a group of sequences (sequences with the same ID are treated as one group) is processed, and transfers the results in the buffer into the overall array. Using the strategy above, which we call it “write through”, we could write back the methylated or non-methylated information back to the overall array and control the usage of memory effectively at the same time. In the end, the overall memory allocating of our program is about: 3GB + 24GB + 1GB = 28 GB (the extra 1 GB is from other static parameters), which is acceptable in general computing platform and proved to be an effective way to optimize the memory allocation.

## Results and Discussions

### Multi-thread parallelized optimization

The basic environment of the computing platform is shown in Table 1.

**Table 1:** Computing environment in the test

Hardware	Index
CPU architecture	x86_64
CPU name	Intel(R) Xeon(R) CPU E5-2670 0 @ 2.60GHz
CPU frequency (MHz)	2593.493
CPU on each node	2*16 cores
Memory on each node (GB)	128
Shared disk (TB)	10

The test consists three main parts: the correctness, the speed-up of parallelism and the scale-up of parallelism.

About the correctness of our program, we checked the results of the test samples by manual work, the results showed complete agreement with the expected results, which means the program could give out the correct results for the given target sequences.

As to tests of speedup, we used the dataset consists of 10,000 lines, 100,000 lines, 1 million lines, 3 million lines and 9 million lines. And we used 6 kinds of different numbers of threads to carry out overall tests. The results of test for 9 million lines of target sequences are shown in Table 2.

**Table 2:** Results for multiple threads

Number of threads	Time (s)	Speed-up
2	37272.4	1.809
4	20259.4	3.548
8	10600.1	7.419
16	5375.5	15.112
32	3072.8	28.008
64	1828.4	47.070

When processing the same amount of data, the serial version of program uses 86062 s. From the comparison between the serial program and the parallel program we can know that our work can achieve a high speedup.

At last, we used 10 million, 50 million and 110 million lines of target sequences to test whether the program will be stable when facing big data to evaluated its scale-up. And the results of these test show the multi-threads program could run normally and keep efficient for big data.

### Multi-processes parallelized optimization

As to the tests for multi-processes program, we used sample 1 and sample 2, whose amount of data are 9 million lines (1087 MB) and 48.492 million lines (59630 MB) respectively. The test results are shown in Table 3.

**Table 3:** Results on multiple computing nodes

Sample	Nodes number	Threads number	Time (s)	Memory allocated (GB)
Sample 1	2	16	840.25	28.9
		32	390.37	28.9
	4	16	468.15	28.9
		32	202.12	28.9
Sample 2	4	32	15806.23	28.9
	6	32	11140.45	28.9

From the lists, we could tell that the program using multi-processes on multiple computing nodes has an obvious speed-up compared to multi-threads only.

### CPU/MIC collaborated parallel computing method

The test computing environment is similar with what we used above, except extra three Intel Xeon Phi coprocessors with 57 cores each. The frequency of every one of these cores is 1.1 GHz. And one of the 57 cores is used as a managing core, so we could use the rest 56 cores.

During the test, we opened two threads on each core, and 112 threads total on each coprocessor. The sample 1 and sample 2 are still 9 million lines and 48.492 lines, respectively. The testing results are shown in Table 4.

**Table 4:** Results for coprocessors

Sample	Number of coprocessor	Thread number	time (s)
Sample 1	1	112	2602
	2	112	1469
Sample 2	1	112	139886
	2	112	70397

From the results, we could figure out that the computing capacity of the whole three coprocessors is about 1.2 times promotion comparing with two CPUs.

## Conclusions

Aiming at a classic problem in epigenetics: the prediction of DNA methylation status, we proposed and implemented a computing tool, and parallelized it with multi-threads and multi-processes. The parallel version with a high speed-up could run both on CPU and Intel Xeon Phi coprocessors. In the program, we implement the prediction of DNA methylation status, threshold filtering, score sharing and result data integration. The pre-process of reference sequence file and concentrated loop IO operation keep the program correct and running in a load balanced and memory efficient way.

In the evaluation part, we evaluate the program about its correctness, its speed-up and scale-up, and analyzed the effectiveness of parallelism. The results of these tests show that the correctness of program is guaranteed both for serial version and parallel version. When we use multiple threads, the speed-up is 47 times with 64 threads, and the speed-up of multiple nodes is nearly linear. Further, we can see that multi-processes on multiple computing nodes, which can be applied to larger dataset, has an obvious speed-up compared to multi-threads only. And the Intel Xeon Phi coprocessors are also used to cooperate with CPUs to achieve a faster computing speed. In general, after parallelism of the program, the running time decreases sharply and the computation resources are fully used.

So far, the program has been paralleled on different levels, taking full use of CPU and Intel Xeon Phi coprocessors on super computer. But as a very important part of the whole pipeline, the program needs data from the upstream. So in the future, we are supposed to combine the whole pipeline of analyzing DNA methylation into one single parallel program to create a seamless tool to analyze DNA methylation.

## Abbreviations

CPU: Central processing unit
DNA: Deoxyribonucleic acid
GPU: Graphics processing unit
MIC: Many integrated core
